# Supplementary material for: Exploring the Biocultural Nexus of Gastrodia elata in Zhaotong: A Pathway to Ecological Conservation and Economic Growth
Source: Biology (Basel). 2025 Jul 11;14(7):846. doi: 10.3390/biology14070846 (PMC12292957; doi:10.3390/biology14070846)
Supplement: Supplementary file 1 [file biology-14-00846-s001.zip › biology-3692375-supplementary.pdf]

Semi-Structured Interview Guide

1. Do you know about *Gastrodia elata* (*Tianma*)? What does this plant look like?
2. How many types of *Tianma* are there locally? What are the differences? Which type is more popular?
3. Where does wild *Tianma* grow (by streams, on slopes, or deep in the forest)? At what elevation?
4. Is *Tianma* cultivated locally? When is it planted? How is it planted?
5. When is *Tianma* harvested? Which part is harvested?
6. What are the traditional uses of *Tianma* in this area? How is it used?
7. Are there any local stories or legends related to *Tianma*?
8. Are there still many people locally who understand the traditional uses of *Tianma*? How is this knowledge passed on?
9. Do local people still harvest wild *Tianma*? Are wild resources still abundant?
10. Is cultivated *Tianma* or wild *Tianma* more popular? Why?
11. Is there a local market for medicinal plants? How is *Tianma* priced?
12. What are the products related to *Tianma* available locally?
13. Has *Tianma* impacted your quality of life? In what ways?
14. How do you protect the environment where *Tianma* grows? Has the government introduced any related policies?
15. Does the local government pay attention to the development of the *Tianma* industry? What measures have been taken?
